# Single-Cell Transcriptomic Changes in Patient-Derived Glioma and U87 Glioblastoma Cell Cultures Infected with the Oncolytic Virus VV-GMCSF-Lact

**DOI:** 10.3390/ijms26146983

**Published:** 2025-07-20

**Authors:** Dmitriy V. Semenov, Natalia S. Vasileva, Maxim E. Menyailo, Sergey V. Mishinov, Yulya I. Savinovskaya, Alisa B. Ageenko, Anna S. Chesnokova, Maya A. Dymova, Grigory A. Stepanov, Galina V. Kochneva, Vladimir A. Richter, Elena V. Kuligina

**Affiliations:** 1 Institute of Chemical Biology and Fundamental Medicine, Siberian Branch, Russian Academy of Sciences, Lavrentyev Avenue, 8, Novosibirsk 630090, Russia; nataly_vas@bk.ru (N.S.V.); yulya_savinovskaya@mail.ru (Y.I.S.); a.ageenko@g.nsu.ru (A.B.A.); a.chesnokova@g.nsu.ru (A.S.C.); maya.a.rot@gmail.com (M.A.D.); stepanovga@niboch.nsc.ru (G.A.S.); richter@niboch.nsc.ru (V.A.R.); kuligina@niboch.nsc.ru (E.V.K.); 2 Sirius University of Science and Technology, Sirius (Federal Territory of Sirius) 354340, Krasnodar Region, Russia; 3 Laboratory of Cancer Progression Biology, Cancer Research Institute, Tomsk National Research Medical Center, Russian Academy of Sciences, Kooperativny Str. 5, Tomsk 634009, Russia; max89me@yandex.ru; 4 Tsivyan Novosibirsk Research Institute of Traumatology and Orthopedics, Department of Neurosurgery, Frunze Street, 17, Novosibirsk 630091, Russia; smishinov@yandex.ru; 5 State Research Center of Virology and Biotechnology “Vector”, Rospotrebnadzor, Koltsovo 630559, Russia; kochneva@vector.nsc.ru

**Keywords:** glioma, glioblastoma, patient-derived cell cultures, virotherapy, vaccinia virus, oncolytic virus, VV-GMCSF-Lact, single cell transcriptome, differentially expressed genes

## Abstract

Oncolytic virotherapy is a rapidly evolving approach to cancer treatment. Our group previously designed VV-GMCSF-Lact, a recombinant oncolytic vaccinia virus targeting solid tumors including gliomas. In this study, we used single-cell RNA sequencing to compare transcriptional responses in human glioma cells, non-malignant brain cells, and immortalized glioblastoma U87 MG cells following infection with this oncolytic virus. We found that proneural glioblastoma cells and microglia-like cells from patient-derived glioma cultures were the most susceptible to VV-GMCSF-Lact. Increased expressions of histones, translational regulators, and ribosomal proteins positively correlated with viral load at the transcript level. Furthermore, higher viral loads were accompanied by a large-scale downregulation of genes involved in mitochondrial translation, metabolism, and oxidative phosphorylation. Levels of early vaccinia virus transcripts are also positively correlated with infection intensity, suggesting that the fate of cells is determined at the early stage of infection.

## 1. Introduction

Adult-type diffuse gliomas are the most common primary brain tumors of the central nervous system [1]. According to the World Health Organization classification, gliomas range from Grade 1 tumors, such as pilocytic astrocytoma, to Grade 4 tumors most commonly represented by glioblastoma [2].

The newest and most potent therapeutic approach to gliomas is immunotherapy, particularly virotherapy [3,4]. Oncolytic viruses can infect malignant cells and trigger their death while also activating an anti-tumor immune response that helps prevent tumor invasion and metastasis [5]. The vaccinia virus (VV) is a promising platform for developing such oncolytic constructs due to its broad cellular tropism as it does not require specific receptors for infection [6]. Targeted viral genome modifications help activate apoptotic/oncolytic processes in cancer cells and engage the immune system, ultimately increasing the effectiveness of virotherapy [7].

We have developed VV-GMCSF-Lact, a recombinant VV derived from the parental Lister strain (L-IVP). In VV-GMCSF-Lact, the thymidine kinase (tk) and vaccinia growth factor (vgf) genes are replaced by human genes encoding granulocyte-macrophage colony-stimulating factor (CSF2) and the apoptosis-inducing protein lactaptin, a fragment of human kappa-casein (CSN3), respectively [8,9]. A phase-one clinical trial of the oncolytic virus VV-GMCSF-Lact in breast cancer patients has been successfully completed, demonstrating the drug’s tolerability, safety, and ability to suppress tumor development (ClinicalTrials.gov ID NCT05376527). Previously, we described how VV-GMCSF-Lact affects a variety of processes in immortalized human glioblastoma U87 MG and U343 MG cells, as well as in patient-derived glioma/glioblastoma and non-malignant human brain cells. By assessing global transcriptome changes at 12/24 h post VV-GM-CSF-Lact infection, we identified differentially expressed genes (DEGs) associated with viral sensitivity/resistance phenotypes [10].

In this study, we used single-cell RNA sequencing to compare shifts in the transcriptome landscapes of human glioma cells, non-malignant brain cells, and immortalized glioblastoma U87 MG cells infected with the oncolytic virus VV-GMCSF-Lact. Assessing mean viral RNA levels, we found that proneural glioblastoma cells are the most sensitive to VV-GMCSF-Lact infection while classical glioblastoma cells show the lowest relative levels of viral transcripts among the cultured human brain cell subtypes under the same infection conditions.

## 2. Results

### 2.1. Cell Cultures and Single-Cell RNA-Seq Data

To investigate how the oncolytic virus VV-GMCSF-Lact affects human brain cells, we used an established glioma model—the immortalized cell line U87 MG (U87)—and patient-derived cultures. The HB1 cell culture was obtained from a patient with gliosis, a non-specific, non-malignant change in glial cells in response to central nervous system damage. The HB3 and HB4 glioblastoma cultures were derived from patient tumors and the NB cell culture was obtained from non-tumor brain tissue during surgery (Table 1).

**Figure 1:**
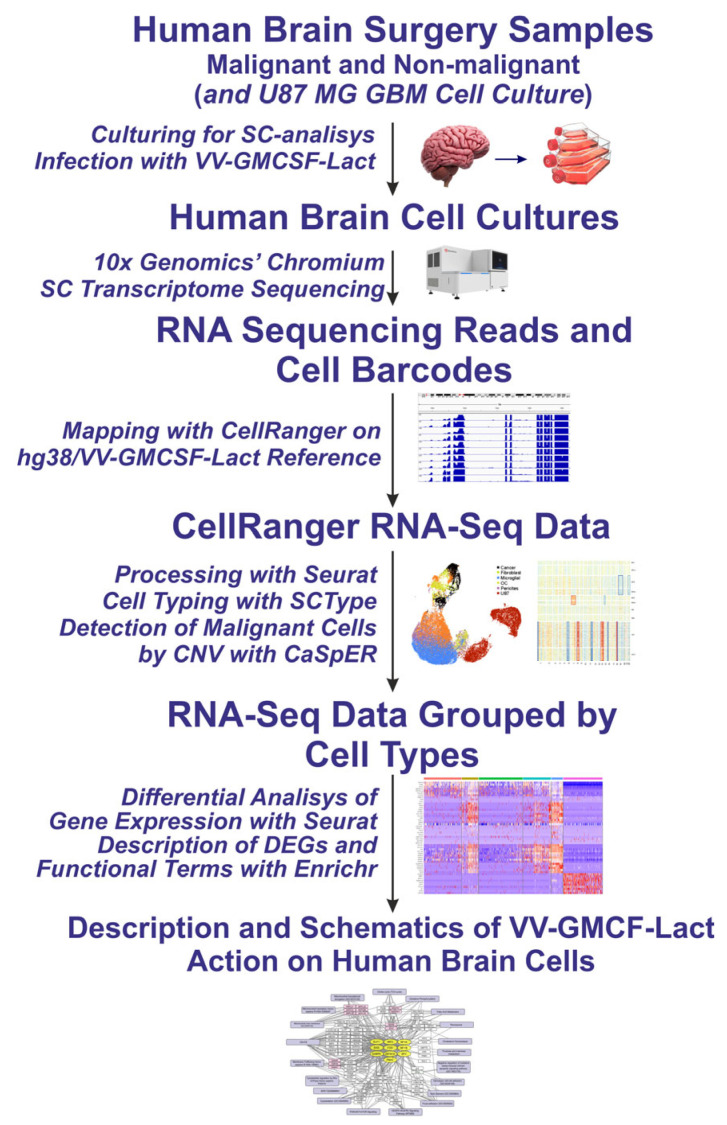
Experimental pipeline from obtaining cultures of malignant and non-malignant human brain cells to single-cell RNA-sequencing, cell-type annotation by RNA patterns, and differential gene expression analysis focusing on cellular response to VV-GMCSF-Lact infection.

Using single-cell transcriptome analysis, we classified human brain cell cultures into known cell types (Figure 1). Cells infected with the VV-GMCSF-Lact virus showed varying relative levels of viral transcripts, indicating distinct subpopulations with differing susceptibility to infection. In contrast, control uninfected cells demonstrated negligible viral transcript reads (Figure S1).

### 2.2. Cell Types Identified Using Single-Cell Sequencing Data

To determine the composition of patient-derived brain cell cultures, we applied the ScType algorithm [11] with a custom database (Tables S1 and S2), which allowed us to classify non-malignant cells into fibroblast-like cells (Fib), microglia-like cells (Mgl), pericytes (Prc), and other cells (OC). Using CaSpER [12], we confirmed that the identified malignant cells had CNV events. Malignant cells from patient-derived HB3 and HB4 cultures were subdivided into classical (CL), proneural (PN), and unclassified GBM (UN) cells (Table S3).

**Figure 2:**
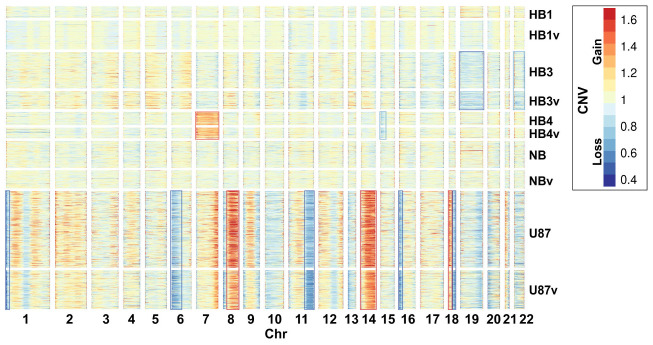
CNV events detected in human brain cell cultures with CaSpER [12]. For comparative analysis, GBM-related subgroups of HB3, HB3v and HB4, HB4v cell cultures were used. The corresponding sets of cells from HB1, HB1v (gliosis) and NB, NBv (normal brain cells) were used as a reference. The most significant CNV events are shown in blue (losses) and red (gains) boxes. The suffix “v” denotes culture samples infected with VV-GMCSF-Lact.

To distinguish between malignant and benign cells, we analyzed copy number variation (CNV) events using CaSpER [12]. Glioblastoma cells in the patient-derived HB3 culture were characterized by the loss of chromosomes 19 and 22 whereas HB4 cells showed the gain of chromosome 7 and loss of 15p. Among the numerous CNV events in U87 cells, the most notable were partial losses in chromosomes 1p, 6p, 11q, 16p, and 18q and gains in chromosomes 8q, 14, and 18p (Figure 2).

**Figure 3:**
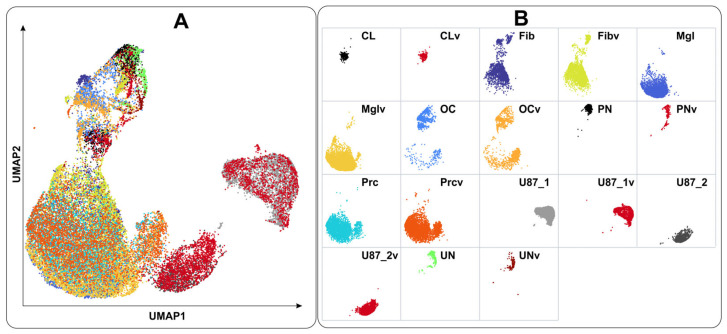
Relationships between single-cell transcriptomes represented as overall (**A**) and detailed (**B**) color-coded UMAP plots. The suffix “v” indicates culture samples infected with VV-GMCSF-Lact.

A variety of genomic aberrations are known, including CNV events, for glioblastomas. For example, aberrations in chr13q, chr19q, and 22q are more commonly found in secondary than in primary glioblstomas [13]. The observed CNV changes suggest that malignant cells in HB3 and HB4 cultures belong to different subtypes of glioblastoma. This is supported by the results of an analysis of a separate glioblastoma single-cell dataset using ScType with custom transcript sets for glioblastoma classification (Table S2). The ScType algorithm classifies the transcriptomes of the HB4 cancer cells as the classical glioblastoma subtype (CL) whereas the HB3 culture contains glioblastoma cells of the proneural subtype (PN) and a comparable number of unclassified (UN), which are neither classical, proneural, nor mesenchymal (Tables S2 and S3, Figure 3).

Aggregated transcriptome data grouped by cell type (Figure S2) are consistent with the results of individual transcriptome analysis. Virus-induced changes are relatively minor compared to differences between cell subtypes, which allows us to reliably distinguish them during VV-GMCSF-Lact infection (Figure 3 and Figure S2).

### 2.3. General Characteristics of the Response of Human Brain Cells to Infection with the Oncolytic Virus VV-GMCSF-Lact

To characterize transcriptome-wide changes induced by VV-GMCSF-Lact infection, we performed differential gene expression analysis. The top 50 genes commonly upregulated across cell types were mostly represented by histone mRNAs (Figure S3, Table S4).

Gene set enrichment analysis revealed that nearly all of the top 10 transcription factors upregulated during VV-GMCSF-Lact infection are involved in modulating histone gene expression and regulating histone modifications (STAT5A, STAT3, TAF7, ATF2, and others in Table S4). Most activated histone genes are located on chromosome 6p22 (Table S4), although not all genes in this locus are induced during infection. This suggests selective viral pressure on host transcription and emphasizes the importance of histone genes in the viral life cycle.

Transcript levels of eight ribosomal proteins and two mitochondrial ribosomal proteins increase during infection (Table S4). These upregulated RPS/RPL genes are associated with the co-translational targeting of newly synthesized proteins to membranes, linking them to the membrane structure and protein secretion. This likely reflects the viral adaptation of the host’s translation machinery to support the synthesis and assembly of new virus particles.

Human genes downregulated by VV-GMCSF-Lact, such as *CSF3, IL1RN, IL24*, and others, are involved in the inflammatory response. At the same time, a separate decrease in the expression of ERK1/2 cascade regulators, which primarily transmit mitogenic signals [14], may contribute to a decrease in the proliferation of infected cells (Table S4).

### 2.4. Viral Transcript Levels Indicate Cell-Type Differences in VV-GMCSF-Lact Susceptibility

**Figure 4:**
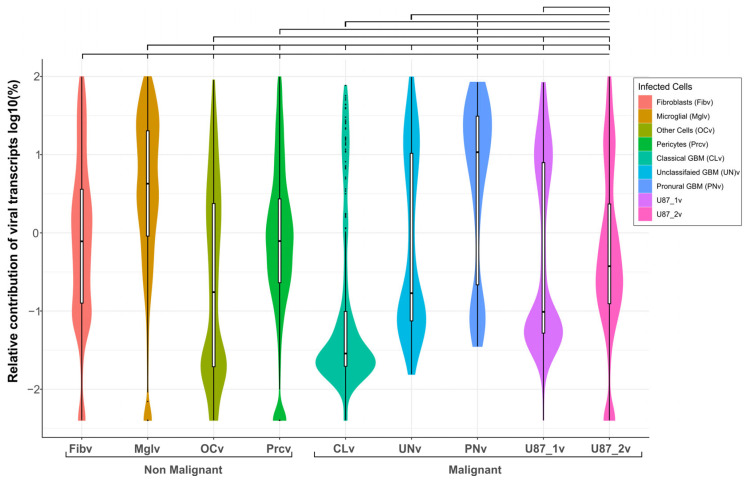
Differences in viral transcript levels across individual cell types in human brain cell cultures after 16 h of VV-GMCSF-Lact infection. Violin and box plots illustrate the relative contribution of viral transcripts to the cellular transcriptome and the median viral transcripts contribution. The top inset shows the significance of the differences in mean values (*t*-test, Holm–Bonferroni adjusted pVal < 0.01, detailed in Table S5). The violin plot is a logarithmic line fit on the x and y axes.

The relative contribution of viral transcripts typically indicates the viral load in infected cells. Comparing these viral RNA levels across cultured cells reveals both their susceptibility to infection and the virus’s impact on cellular processes. Among human brain cell subtypes analyzed in vitro, proneural GBM cells were most susceptible to VV-GMCSF-Lact infection while classical GBM cells showed the lowest viral transcript levels under the same conditions (Figure 4, Table S5). Overall, the susceptibility of cells to VV-GMCSF-Lact infection followed this order: PNv > Mglv >> UNv > Fibv > U87_1v > U87_2v > Prcv >> OCv > CLv (Table S5).

Microglia and astrocytes have been shown to attenuate oncolytic virus replication in mouse glioma by acting as VV traps in vivo [15]. Our data confirm that microglia are among the most susceptible to VV-GMCSF-Lact infection in human brain cell cultures in terms of mean relative viral transcript levels at 16 h post infection. In addition, proneural GBM cells demonstrate significantly higher susceptibility than microglia based on mean viral transcript levels (Figure 4, Table S5).

### 2.5. Human Brain Cell Transcripts Associated with Resistance or Susceptibility to VV-GMCSF-Lact

To pinpoint genes associated with cellular susceptibility or resistance to VV-GMCSF-Lact infection, we analyzed the correlation between individual host mRNAs and mean viral transcript levels across the identified brain cell types (Figure S4). Differential gene expression was therefore assessed in relation to the cell-type-specific susceptibility of cultured cells to infection.

**Figure 5:**
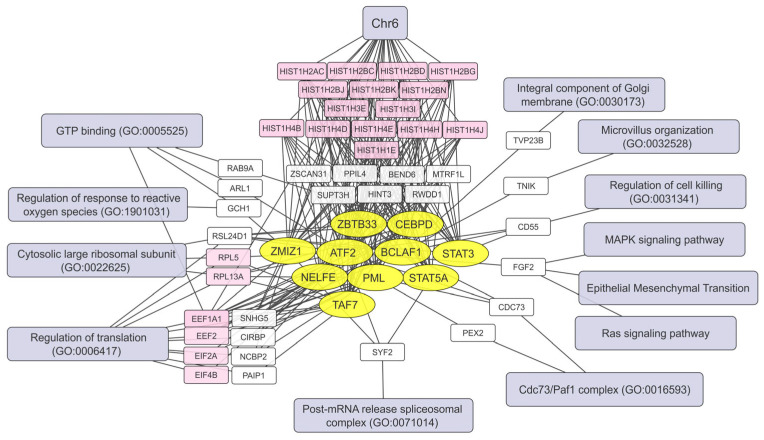
Transcriptional networks activated by VV-GMCSF-Lact infection. The network illustrates the relationships between transcription factors (yellow ovals), activated genes (white and pink rectangles), and associated cellular processes (light gray–blue). The analysis is based on transcripts showing a positive correlation (Pearson’s R > 0.5) with the mean VV-GMCSF-Lact RNA levels in infected cells grouped by type. Genes with similar functions are highlighted in pink.

**Figure 6:**
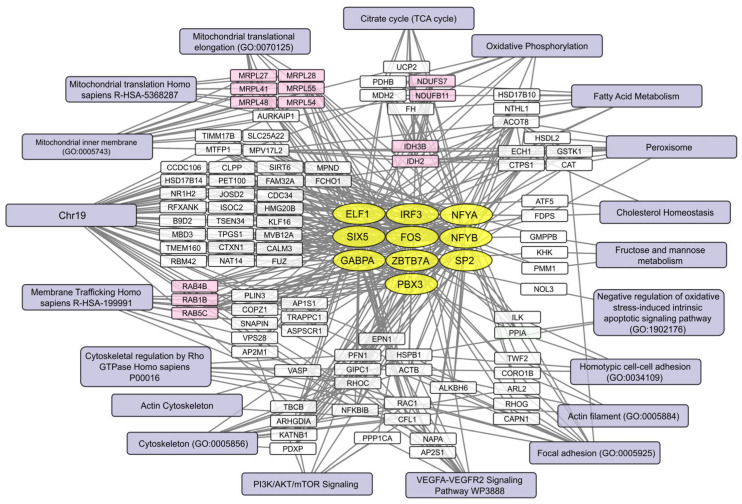
Transcriptional networks suppressed by VV-GMCSF-Lact infection. The network illustrates the relationships between transcription factors (yellow ovals), downregulated genes (white and pink rectangles), and associated cellular processes (light gray–blue). The analysis is based on transcripts showing a negative correlation (Pearson’s R < −0.5) with the mean VV-GMCSF-Lact RNA levels in infected cells grouped by type. Genes with related functions are highlighted in pink.

Based on Pearson’s correlation coefficient, we selected the top 300 transcripts positively correlated (R > 0.5) and the top 300 transcripts negatively correlated (R < −0.5) with the mean viral transcript contribution. These transcript lists were analyzed using Enrichr to identify common characteristics such as transcription factors, key biological processes, chromosomal localization, etc. (Figure 5 and Figure 6).

The largest functional group shown in Figure 5 consists of histone mRNAs encoded on chromosome 6, which are commonly induced when cells are infected with the virus (Figure S3 and Table S4). Thus, the increase in histone gene expression during VV-GMCSF-Lact infection is not just a general characteristic of cell populations but also reflects viral burden.

The expression of genes involved in translation initiation (*EIF2A, EIF4B*) and elongation (*EEF1A1, EEF2*), as well as genes of ribosomal proteins (*RPL5, RPL13A*), directly correlates with the mean viral transcript levels in infected cells (Figure 5). Therefore, these genes and their products can be considered both infection markers and modulators of cellular processes that enable viral replication, the assembly of new viral particles, and the spread of VV-GMCSF-Lact infection.

Human brain cell transcripts inversely correlating with mean VV-GMCSF-Lact RNA levels fall into three diverse mRNA groups.

The first group includes factors associated with mitochondria (Figure 6): mRNAs encoding proteins of mitochondrial ribosomes (MRPL27, -28,-41,-48,-54,-58), components of the respiratory chain (NDUFS7, NDUFB11), mitochondrial isocitrate dehydrogenases (IDH2, IDH3B), and others. Transcripts encoding mitochondrial proteins are depleted in infected cells, which directly indicates a large-scale suppression of translation, metabolism, and oxidative phosphorylation in mitochondria.

Genes on chromosome 19 encode a second group of mRNAs with reduced expression corresponding to higher viral transcript levels (Figure 6). As shown in Section 2.2, the lower expression of chromosome 19 transcripts can be linked to the CNV events in the patient-derived HB3 glioblastoma cell culture (Figure 2).

Finally, a group of transcripts encoding components of the cytoskeleton, actin filaments, and cell adhesion molecules is also downregulated as viral RNA levels increase (Figure 6).

Taken together, our data highlight the central role of histone gene activity, translation machinery genes, mitochondrial gene transcripts, transcripts of chromosome 19, and cytoskeletal transcripts in the cellular response to VV-GMCSF-Lact. The modulation of these processes is directly linked to both viral infection and the oncolytic action of the virus.

### 2.6. Pre-Infection Transcript Signatures Linked to VV-GMCSF-Lact Susceptibility in Human Brain Cells

One of the most important questions regarding the effect of the virus on cells is what determines their susceptibility to infection. To explore this, we analyzed transcripts in uninfected cells whose levels were either positively or negatively correlated with viral RNA levels in the corresponding VV-GMCSF-Lact-infected cells. Essentially, we used the same approach as before—identifying transcripts that correlate with viral RNA in infected cells—but applied it to their uninfected counterparts (Figure S5).

Transcripts with elevated levels before infection, which are associated with higher viral RNA levels in corresponding infected brain cells, mostly cluster within a few relatively small groups linked to components of the innate immune system. In addition, transcripts involved in epithelial–mesenchymal transition, hypoxia, and apoptosis may also be associated with viral RNA levels after infection (Figure S6).

Notably, the individual factors and processes that predispose cells to VV-GMCSF-Lact infection showed little overlap with those activated by the virus during infection (Figure 5 and Figure S6). CD55 (complement accelerating factor) and FGF2 (fibroblast growth factor 2) were two genes whose increased expression correlated with viral RNA levels both before and after infection. Both CD55 and FGF2 are known to be involved in tissue remodeling and protection, the regulation of fibroblast activity, and cancer development and immune evasion [16,17]. None of the top 10 transcription factors positively correlated with viral RNA levels in uninfected cells were shared with those in infected cells (Figure 5 and Figure S6).

In contrast, transcripts that were expressed at lower levels in uninfected cells or showed a negative correlation with viral load had more shared features. In infection-susceptible cells, genes regulated by the transcription factors NFYA/NFYB, ZBTB7A, and PBX3 were downregulated both in the presence and absence of infection. The reduced expression of transcripts encoding focal adhesion proteins and nuclear transcripts for mitochondrial ribosomal proteins and oxidative phosphorylation factors was associated with higher levels of viral mRNA in infected and matched uninfected cells (Figure 6 and Figure S7).

Of particular interest was the inverse correlation between the reduction of chr19-encoded transcripts and the mean VV-GMCSF-Lact RNA level in both infected and corresponding uninfected cells (Figure 6 and Figure S7). As shown in Figure 2, glioblastoma HB3 cells demonstrated CNV losses in chromosome 19 and represented the only culture in which the proneural subtype was identified (Table S3). Proneural cells were the most susceptible to infection based on mean VV-GMCSF-Lact transcript levels (Section 2.4, Figure 4, Table S5). Thus, genomic aberrations on chromosome 19 may have contributed to the increased susceptibility of the proneural subtype of glioblastoma to the oncolytic virus.

### 2.7. VV Early Genes Are the Primary Viral Transcripts Linked to Infection Severity

When analyzing VV-GMCSF-Lact transcripts for correlations with mean viral transcript levels across different human brain cell subtypes at 16 h post infection, we found no viral transcripts that negatively correlated with total viral RNA (Pearson’s correlation coefficient R < −0.5). It should be noted, that previous “bulk” transcriptome analyses have shown that VV-GMCSF-Lact infects both normal and malignant cells, similarly expresses the entire pool of viral transcripts, and replicates in both malignant and non-malignant cell types [10].

The list of viral transcripts that positively correlated with the mean viral RNA percent is presented in Table 2. It includes factors that participated in or regulated vital cellular processes, such as F1L—large subunit of mRNA capping enzyme and transcription termination factor, N1L—an inhibitor of both apoptosis and NF-κB activation, J4R—DNA-dependent RNA polymerase subunit, and others (Figure S8, Table 2).

For viral transcripts that positively correlate with the susceptibility of human brain cells to infection, the most characteristic feature is their expression stage. Among the 13 differentially expressed viral mRNAs with known expression stages, nine represent “early” transcripts while only two are “late” ones (Table 2). Thus, it can be suggested that early VV-GMCSF-Lact transcripts (such as F1L, D4R, and J3R) make a significant contribution to overcoming host cell response by this oncolytic virus.

## 3. Discussion

Oncolytic virotherapy is a rapidly developing field focused on suppressing the viability of cancer cells, activating an immune response against malignancies, and preventing metastasis. The vaccinia virus (VV) is one of the most extensively studied platforms for developing oncolytic treatments [30]. The VV is a dsDNA virus of the *Poxviridae* family and its ~195 kb genome consists of approximately 250 genes [31,32]. The virus enters cells by either micropinocytosis or direct fusion with the plasma membrane [33]. Unlike many dsDNA viruses replicating in the host nucleus, poxviruses encode their own replication machinery and replicate in the cytoplasm. Viral genes are expressed in a biphasic manner, with early genes encoding non-structural proteins involved in genome replication and late genes encoding structural proteins [34].

VV infection triggers widespread changes in the molecular and genetic landscape of the cell. This can either suppress viral activity or promote extensive viral replication, leading to cell death. A previous study using deep RNA sequencing on VV-infected HeLa cervical cancer cells identified upregulated host RNAs linked to NF-kappa B signaling, apoptosis, signal transduction, and ligand-mediated signaling, likely reflecting the cellular response to viral invasion [35].

We developed a promising recombinant vaccinia virus—VV-GMCSF-Lact—for the treatment of solid tumors, including glioma [8,9], and assessed how it affects human cells using immortalized and patient-derived glioma cultures [10]. Examining ‘bulk’ transcriptome changes in host cells 12 and 24 h after infection, we found a general upregulation of histone genes and higher expression of genes associated with the interferon-gamma response, NF-kappa B signaling, and inflammation mediated by chemokine and cytokine signaling (among others, as detailed in [10]). By contrast, genes involved in cell cycle progression, including spindle organization, sister chromatid segregation, and the G2/M checkpoint, were generally downregulated in glioma cells upon infection with VV-GMCSF-Lact. The increased expression of genes responsible for Golgi vesicle formation, protein transport, and secretion correlated with the cytotoxic dose of the virus (CD_50_). The upregulation of genes encoding proteins that participate in the maturation of pol II nuclear transcripts and mRNA splicing was associated with increased sensitivity to viral cytotoxicity [10].

This paper has presented a single-cell RNA-seq analysis of transcriptomic changes associated with VV-GMCSF-Lact infection (Figure 1, Table 1). The single-cell approach enabled us to deconstruct ‘bulk’ transcriptomes into cell-type-specific datasets in order to analyze RNA expression patterns within distinct cell populations (Table S3). To classify single-cell RNA-seq data according to cell types, we applied the ScType algorithm [11] and validated the classification of individual cells from personalized cell cultures as malignant or non-malignant using detailed CNV analysis with CaSpER [12] (Figure 2 and Figure 3).

According to the molecular signature, GBM can be classified into 3 subtypes: proneural, classical and mesenchymal [36]. The frequency of these subtypes varies between studies and can be estimated approximately as: classical subtype (~20%), proneural (~33%), or mesenchymal (32%) [37]. The remaining 15% can be considered unclassified or mixed-type glioblastomas.

ScType was used to categorize glioblastoma cells into classical, mesenchymal, and proneural subtypes (Table S2). No mesenchymal subtype cells were detected in either of the analyzed glioblastoma cultures (HB3 and HB4). Cells that did not fit into the classical, proneural, or mesenchymal subtypes were considered unclassified GBM cells (Table S3, Figure 2 and Figure 3).

Non-malignant human brain cells in patient-derived cell cultures were classified as fibroblast-like, microglia-like, pericyte-like, and other cell types (Table S3, Figure 2 and Figure 3). Different cell types in patient-derived cultures demonstrated distinct transcriptomic changes in PCA and UMAP coordinates after viral infection (Figure 3 and Figure S2), directly indicating fundamental differences in how human brain cells respond to VV-GMCSF-Lact.

Our data suggests that the viral modulation of cellular transcription, controlled by STAT5A, TAF7, ATF2, and other transcription factors, primarily targets the expression of multiple histone genes. Many, but not all, of these upregulated histone genes are located on chromosome 6p22 (Table S4).

VV-GMCSF-Lact infection broadly increased the mRNA levels of several ribosomal proteins, particularly those involved in the co-translational targeting of nascent proteins to the membrane, suggesting a role in facilitating viral assembly and invasion. This may indicate that the virus is adapting the host translation machinery to support the synthesis and assembly of new viral particles. Concurrently, transcripts associated with immune response, including genes in the ‘inflammatory response’, ‘TNF-alpha signaling via NFkB’, and ‘interferon alpha response’ groups, were downregulated (Table S4).

VV-GMCSF-Lact also affected the ERK1/2 kinase signaling cascade. It is typically activated by mitogens, growth factors, and G protein-coupled receptors to promote cell proliferation, differentiation, and development [14]. The ERK1/ERK2 pathway genes were downregulated in infected cells, indicating that viral factors can suppress proliferation and growth in human brain cells (Table S4).

To assess the susceptibility of individual human brain cell types to VV-GMCSF-Lact infection, we analyzed the relative levels of viral transcripts. Based on the mean contribution of viral transcripts 16 h post infection, cell types can be ranked as follows: PNv > Mglv >> UNv > Fibv > U87_1v > U87_2v > Prcv >> OCv > CLv. Proneural glioblastoma and non-malignant microglial cells expressed the highest levels of viral transcripts whereas classical glioblastoma cells had the lowest under the same conditions (Figure 4, Table S5). Some oncolytic viruses have been demonstrated to disrupt crosstalk between the tumor and its microenvironment during virotherapy. For example, the herpes virus can accumulate in microglia but may be inhibited through the STAT1-interferon pathway [38]. Similarly, Kober and colleagues reported the attenuation of the replication of vaccinia virus LIVP in microglial cells and astrocytes [15]. Our data indicate that transcriptionally active VV-GMCSF-Lact successfully accumulates in microglial cells, suggesting it may prove more effective in immunosuppressive microenvironments through activating anti-tumor immune mechanisms.

To explore the processes underlying differential responses of human brain cells to VV-GMCSF-Lact infection, we analyzed correlations between individual RNA levels and the mean viral transcript content in specific cell types (Figure S4). The increase in histone mRNA levels (mainly encoded on chromosome 6), as well as transcripts involved in translation and its regulation, points to a key role of histones and the translational machinery in determining the susceptibility of human brain cells to infection (Figure 5). These findings also suggest selective pressure exerted by the virus on host transcription and highlight the importance of histone genes in supporting the viral life cycle. Additionally, the virus appears to modulate transcription factors STAT3/STAT5, NELFE, ATF2, TAF7, and others (Figure 5), enhancing cellular response to infection.

A subset of host mRNAs showed a consistent negative correlation with mean VV-GMCSF-Lact RNA levels across all human brain cell types analyzed. These included transcripts of mitochondrial proteins, membrane transport components, cytoskeletal elements, and others (Figure 6). This suggests that mitochondrial translation, oxidative phosphorylation, and mitochondria-associated metabolic processes are significantly disrupted by VV-GMCSF-Lact infection.

Importantly, a distinct group of chr19 genes was also downregulated as viral RNA levels increased in both infected and corresponding non-infected cells of different subtypes (Figure 6 and Figure S7). In the patient-derived HB3 cell culture, the decreased expression of chr19 genes was identified as a CNV event (Figure 2). This culture also contained proneural glioblastoma cells, which demonstrated the highest sensitivity to the virus infection based on mean viral RNA levels (Figure 4, Table S5). Therefore, the partial loss and/or transcriptional regulation of chromosome 19 may be a significant factor underlying the increased sensitivity of proneural glioblastoma cells to oncolytic virus infection. Additionally, the virus suppressed transcription factors from the IRF-, NFY-, and FOS- families (among others in Figure 6), further increasing cellular sensitivity to infection.

Viral transcripts that positively correlated with mean VV-GMCSF-Lact RNA levels in cultured host cells were mainly expressed from ‘early’ genes (Figure S8, Table 2). It is known that VV-GMCSF-Lact gene expression occurs in three phases—early (2 h), intermediate (4 h), and late (6 h)—with the full replication cycle completed in ~6 h [34].

Although our single-cell data were collected after 16 h of incubation and captured transcripts from all stages, only a subset of early viral genes showed a correlation with infection intensity across cell groups. This enrichment suggests that early viral gene expression plays a key role in determining cellular susceptibility. In both cancerous and non-cancerous cells, this likely reflects the transcriptional state of each cell and its ability to respond to a cytolytic infection at an early stage. To investigate this, we correlated pre-infection transcript levels with viral RNA abundance in the same cell types post infection. Our findings showed that the downregulation of genes controlled by the transcription factors NFYA/NFYB, ZBTB7A, and PBX3 was a common feature of cells susceptible to infection both before and after exposure (Figure 6 and Figure S7).

Thus, our transcriptomic analysis revealed a correlation between pre-existing chromosomal aberrations in glioblastoma and the efficacy of infection with the oncolytic virus VV-GMCSF-Lact in patient-derived glioblastoma cultures, non-malignant brain cells, and the immortalized U87 MG glioblastoma cell line. We described the changes to individual transcripts and their functional groups, providing a mechanistic basis for understanding of VV oncolysis. These findings not only shed light on the fundamental processes of infection with VV-GMCSF-Lact but can also inform the development of next-generation immunotherapies and help establish personalized virotherapy strategies guided by tumor genetics.

## 4. Materials and Methods

### 4.1. Immortalized Human Glioblastoma Cell Line U87 MG

The human U87 MG cell line was purchased from the Russian cell culture collection (Russian Branch of the ETCS, St. Petersburg, Russia). The cells were cultivated in Minimum Essential Medium α (MEM α; Sigma-Aldrich, St. Louis, MO, USA) supplemented with 10% FBS (Gibco BRL Co., Gaithersburg, MD, USA), 2 mM L-glutamine (Sigma-Aldrich, St. Louis, MO, USA), 250 mg/mL amphotericin B, and 100 U/mL penicillin/streptomycin (Gibco BRL Co., Gaithersburg, MD, USA) at 37 °C in a humidified atmosphere containing 5% CO_2_. Genetic identification of the U87 MG cell line was performed using the COrDIS Plus kit (GORDIZ, Moscow, Russian Federation). The STR profile of U87 MG corresponded to those published in international ATCC and DSMZ databases.

### 4.2. Patient-Derived Cell Cultures

Glioma tissue samples were obtained at the Novosibirsk Research Institute of Traumatology and Orthopedics (Novosibirsk, Russia) from patients who provided informed consent. A sample of normal brain tissue (NB) was collected from a patient without malignant tumors at the time of surgery (see Table 1 for details). The study was approved by the Committee on the Ethics of the Novosibirsk Research Institute of Traumatology and Orthopedics (protocol no. 050/17 68 of 11 September 2017).

Patient-derived cell cultures were processed as described previously [10]. Briefly, human brain samples were mechanically dissociated in Iscove’s Modified Dulbecco’s Medium (IMDM, Sigma-Aldrich, St. Louis, MO, USA), washed with a 10x excess of phosphate-buffered saline (PBS), and pelleted by centrifugation at 300× *g*. The cells were then seeded in 6-well plates with IMDM supplemented with 10% FBS, 2 mM L-glutamine, 100 U/mL penicillin, 100 µg/mL streptomycin, and 250 mg/mL amphotericin B. The plates were incubated at 37 °C in a humidified atmosphere containing 5% CO_2_ for cell adhesion. After reaching 70–80% confluence, the cells were harvested using TripLE-Express (GIBCO, Thermo Fisher, Waltham, MA, USA) and subcultured for single-cell analysis.

### 4.3. Oncolytic Virus VV-GMCSF-Lact

VV-GMCSF-Lact was engineered from the VV Lister strain (L-IVP). The viral thymidine kinase (tk) and virus growth factor (vgf) gene fragments were substituted with the human GM-CSF gene (*CSF2*) and the pro-apoptotic fragment of human kappa-casein gene (*CSN3*), respectively [8]. VV-GMCSF-Lact was produced in African green monkey kidney cells 4647 and purified as described in [39]. The viral titer was determined using a plaque-forming assay and expressed as the number of plaque-forming units per volume (PFU/mL) [39].

### 4.4. Treatment with VV-GMCSF-Lact for Single-Cell RNA Sequencing

Cells of immortalized (U87 MG) and patient-derived (HB1, HB3, HB4, NB) cultures were plated into 6-well plates (1 million cells/well) and treated with 0.1 PFU per cell of VV-GMCSF-Lact. After 16 h incubation at 37 °C in a humidified atmosphere containing 5% CO_2_, cells were harvested using Triple-Express (GIBCO, Thermo Fisher, Waltham, MA, USA). Cells incubated in the same conditions but without exposure to the virus were used as the control.

### 4.5. Single-Cell RNA Sequencing

Cell counting and viability assay were carried out using 0.4% trypan blue (Thermo Fisher Scientific, USA) and an automated cell counter Luna II (Logos Biosystem, Anyang-si, Republic of Korea). Cell suspensions were diluted to 1000 cells/mL, with each sample not exceeding 10,000. Single-cell RNA sequencing was performed on the 10x Genomics Chromium Controller using the Single Cell 3′ Reagent Kit v3.1 (10x Genomics, Pleasanton, CA, USA). cDNA amplification and library construction were carried out according to the manufacturer’s protocol. The concentration of cDNA libraries was measured using the dsDNA High Sensitivity kit on a Qubit 4.0 fluorometer (ThermoFisher Scientific, Waltham, MA, USA), followed by quality assessment with the High Sensitivity D1000 ScreenTape on a 4150 TapeStation (Agilent, Santa Clara, CA, USA). cDNA libraries were pooled, denatured, and sequenced using Genolab M (GeneMind, Shenzhen, China) with pair-end reads: 28 cycles for read 1, 90 cycles for read 2, and 10 cycles for i7 and i5 indexes. Sequencing was carried out at the Medical Genomics Center of Collective Use at the Tomsk National Research Medical Center (Russia).

### 4.6. Single-Cell Transcriptome Analysis

Raw sequencing reads were mapped to the human genome (GRCh38/hg38) with the addition of the VV-GMCSF-Lact genome sequence as a separate chromosome. The *CSF2* and *CSN3* lactaptin gene sequences were excluded from the human genome by masking the corresponding regions with N. Reads were mapped with Cellranger 6.1.2 and sequencing mapping data were analyzed with Seurat (R 4.4.0) [40]. To resolve the cellular composition of patient-derived human brain cell cultures, we applied the ScType algorithm [11] with the custom table of cell markers that were optimized using Enrichr libraries “CellMarker Augmented 2021” and “PanglaoDB Augmented 2021” (Tables S1 and S2) [41,42]. Differential gene expression analysis was performed using either Seurat or DESeq2 1.36.0 (aggregated SC data) as indicated in the figure and table legends. Gene enrichment analysis of lists of upregulated and downregulated genes was performed with Enrichr in RStudio 9.4.191329 [43]. Schemas were created with Cytoscape 3.10.3.

## Figures and Tables

**Table 1:** General characteristics of patients and cell cultures.

Cell Culture	Gender	Age (Years)	Diagnosis	IDH1 *	Ref **
HB1	female	32	Gliosis	– ***	–
HB3	male	34	GBM	wt	–
HB4	female	47	GBM	wt	–
NB	female	74	–	–	[10]
U87	male	44	GBM	wt	[10]

*—status of the *IDH1* gene mutations; Wt—wild type. **—previously reported bulk transcriptome data. ***—no relevant data (or references).

**Table 2:** VV-GMCSF-Lact transcripts that directly correlate with the mean levels of viral transcripts in cultured human brain cells at 16 h post infection.

Vir Gene	Pearson Correlation Coefficient ^(1)^	Temporal Expression ^(2)^	Preferred Name ^(2)^. Annotation	Ref
F1L	0.68	Early	Hypothetical protein. The large subunit of mRNA capping enzyme. Transcription termination factor	[18]
F10L	0.67	Late	Ser/Thr kinase	[19]
C4L	0.66	Early/Late	Hypothetical protein	– ^(3)^
N1L	0.66	Early/Late	Putative virulence factor. N1 inhibits both apoptosis and NF-κB activation	[20]
D4R	0.64	Early	Temporal expression. Uracil–DNA glycosylase	[21]
E4L	0.63	Early	RNA polymerase subunit	–
J4R	0.63	Early	DNA-dependent RNA polymerase subunit rpo22	–
F16L	0.61	–	Hypothetical protein. Recombinase superfamily	[22]
N2L	0.59	Early	Putative alpha aminitin-sensitive protein	–
D1R	0.59	Early	Large subunit of mRNA capping enzyme	–
F14.5L	0.58	–	Hypothetical protein	–
174	0.58	–	–	–
C12L	0.58	Early	Serine protease inhibitor-like SPI-1	[23]
F11L	0.57	–	Hypothetical protein. Inhibition of RhoA signaling	[24]
201	0.57	–	–	–
D7R	0.57	Early	DNA-dependent RNA polymerase subunit rpo18	[25]
D11L	0.57	Late	ATPase, nucleoside triphosphate phosphohydrolase-I, NPH-I. Similar to VACCP-D11L; transcription elongation, termination, release factor	[26]
H6R	0.56	–	DNA topoisomerase type I	[27]
J3R	0.56	Early	Poly(A) polymerase small subunit	[28]
K6L	0.56	–	Putative monoglyceride lipase	[29]

^(1)^—Correlation with the mean VV-GMCSF-Lact transcript level in human brain cells. ^(2)^—Temporal expression, preferred names, and annotations of VV genes from the NCBI Gene database (https://www.ncbi.nlm.nih.gov) accessed on 8 December 2024. On the basis of previous bulk transcriptomic analyses it can be concluded that VV infects both normal and malignant cells, similarly expresses the entire pool of viral transcripts, and replicates in both cell types. ^(3)^—Data not available.

## Data Availability

All data supporting the findings of this study are available in the article and supplementary files.

## Supplementary Materials

The following supporting information can be downloaded at: https://www.mdpi.com/article/10.3390/ijms26146983/s1.

## Abbreviations

CD_50_	cytotoxic dose
CSN2	kappa-casein gene
DEG	differentially expressed gene
GBM	glioblastoma, previously referred to as ‘glioblastoma multiforme’
GM-CSF	granulocyte-macrophage colony-stimulating factor (product of the CSF2 gene)
HC	hierarchical clustering
NB	normal brain tissue cell culture
PCA	principal component analysis
PFU	plaque-forming unit
tk	thymidine kinase
U87	U87 MG cell culture
VV	vaccinia virus
vgf	virus growth factor
VST	variance stabilizing transformation
