# Supplementary material for: Single-Cell Transcriptomic Changes in Patient-Derived Glioma and U87 Glioblastoma Cell Cultures Infected with the Oncolytic Virus VV-GMCSF-Lact
Source: Int J Mol Sci. 2025 Jul 20;26(14):6983. doi: 10.3390/ijms26146983 (PMC12295809; doi:10.3390/ijms26146983)
Supplement: Supplementary file 1 [file ijms-26-06983-s001.zip › ijms-3745520-supplementary.pdf]

Table S1. Gene sets (cell markers) used to annotate cell types across the entire single-cell RNA-seq dataset with ScType. These gene sets were optimized using Enrichr libraries "CellMarker Augmented 2021" and "PanglaoDB Augmented 2021".

cellName	geneSymbolmore1
Endothelial	PECAM1,VWF,A2M,APOLD1,FLT1,TM4SF1,CD34,MCAM,ENG,PTPRC,VCAM1,CDH5
Pericytes	ACTA2,TAGLN,CALD1,TPM2
Cancer	HSPA9,SUPT16H,ZWILCH,CSE1L,ACTL6A,PA2G4,EIF2S1,IPO7,PAICS,LRPPRC,HSP90B1,IPO5,CCT6A,SYNCRIP,CAPRIN1
Microglial	TPT1,CSF1R
Brain_Pericyte	HEYL,MUSTN1,OLFML2A
Monocyte	CEBPB,CXCL8,BTG1,IL1B,GLRX,SOD2,SAT1,IER3
Fibroblast	COL1A1,COL3A1,COL1A2,FN1

Table S2. Gene sets (cell markers) used to annotate the GBM subtypes of selected GBM cells in the single-cell RNA-seq dataset with ScType. These gene sets were optimized using Enrichr libraries "CellMarker Augmented 2021" and "PanglaoDB Augmented 2021".

cellName	geneSymbolmore1
CL	NOTCH3,LFNG,RB1,JAG1,CCND2,CDKN2A,SMO,CDK4,GAS1,NES,GLI2,EGFR
MS	TRADD,NF1,PTEN,MET,MERTK,CHI3L1,RELB,TNFRSF1A,CD44
PN	IDH1,PDGFRA,SOX2,PIK3R1,PIK3CA,NKX2-2,DCX,OLIG2,TCF4,DLL3,ASCL1

Table S3. Cellular composition of patient-derived human brain cell cultures and the U87 cell line. Cellular composition was defined using the ScType algorithm with an optimized table of GBM subtype transcriptome markers from the 'Elsevier Pathway Collection' Enrichr library. In the case of the U87 GBM line, we identified two distinct cell subpopulations, named U87_1 and U87_2.

Type	Cell Types or GBM Subtypes[(1)]	Cell culture										Infected/ Not Infected[(3)]
		HB1	HB1v	HB3	HB3v	HB4	HB4v	NB	NBv	U87	U87v	
		Relative Cell Content, % [(2)]										
Non-malignant	Fib	31.88		9.34		27.89		18.59				0.90
	Fibv		25.69		21.73		29.75		2.18			
	Mgl	21.93		34.97		18.65		16.90				1.57
	Mglv		31.43		40.25		32.47		40.72			
	OC	13.56						58.12				0.77
	OCv		16.77						38.68			
	Prc	32.62		33.92		35.87		6.40				0.92
	Prcv		26.16		28.59		26.57		18.43			
Malignant (GBM)	CL					17.63						0.61
	CLv						10.38					
	UN			8.86		0.37						0.47
	UNv				3.90		0.42					
	PN			12.96		0.16						0.45
	PNv				5.53		0.42					
	U87_1									76.36		0.56
	U87_1v										42.40	
	U87_2									23.64		2.44
	U87_2v										57.65	
**Total number of cells**	**30,557**	**2,161**	**4,340**	**4,174**	**4,917**	**1,893**	**2,390**	**1,657**	**2,252**	**3,714**	**3,059**	--

[(1)] Fib – fibroblast-like cells; Mgl – microglia-like cells; Prc – pericytes; OC – other non-malignant brain cells; CL – classical GBM subtype; PN – proneural GBM subtype; UN – GBM cells with unclassified subtype. The suffix "v" denotes cell culture samples infected with VV-GMCSF-Lact.

[(2)] Percentage of cells in the subpopulation relative to the total cell count in the sample.

[(3)] Ratio of the combined percentages of cell subtypes in virus-infected and corresponding uninfected samples.

Note that the analysis of cell types (except U87 MG) is based on different cell cultures. Thus, this diversity itself constitutes biological replicates for analysis, inferences and conclusions.

Table S4. Gene set enrichment analysis of transcripts upregulated or downregulated after 16 hours of VV-GMCSF-Lact infection in human brain cells. Differentially expressed transcripts were identified using DeSeq2 analysis of infected vs control cells, sorted avg_log2FC, and filtered by p_val_adj < 0.05. Selected results from the top 300 upregulated and downregulated gene sets in Enrichr are shown.

Enrichr Library	Term	Adj p_val(1)	Combined Score(1)	Representative Genes(2)
**Upregulated**				
ENCODE and ChEA Consensus TFs from ChIP-X	STAT5A ENCODE	5.9E-09	163	HIST1H2BO(16);MAP1LC3B2;HINT3;SYF2;HYI;ZNF778
	TAF7 ENCODE	3.6E-08	82	HIST1H2BO(17);NDUFB1;UNK;RPL38;RPL37;RRP15;SNHG8;SNHG9;MRPL30;RPS28
	ATF2 ENCODE	1.8E-06	36	GALNT11;HIST2H2AB(26);IFT52;FRAT2;HINT3;RPL38;RPL37;SOX4;RRP15;PAAF1;RMRP;EPM2A;SNHG8;SNHG9
	NELFE ENCODE	2.5E-06	87	HIST1H2BO(12);PIK3R3;SNHG9;RPS28;RPS27;CALM2
	ZMIZ1 ENCODE	2.6E-06	46	HIST1H2BO(16);RPL36A;ACAT2;DCAF17;HINT3;CHCHD7;ZRSR2;FBXW11;SNHG8;UBE2E1
	STAT3 ENCODE	9.5E-06	44	RNASEL;PHLPP1;HIST1H2BO(15);DNAJC24;WDR70;RPL38;NEK8;MKKS;GIN1
	BCLAF1 ENCODE	2.0E-04	29	HIST1H2BO(15);RPL36A;WDR70;HINT3;SLC25A40;RPL37;EIF5B;GIN1;AP2B1;PRKAB1;MRPL24
	PML ENCODE	3.8E-04	22	HIST2H2AB(18);RPL36A;PIK3R3;DCAF17;CHCHD7;SLC25A40;RPL38;TAF11;SNHG8;SNHG9
	ZBTB33 ENCODE	7.1E-04	25	HIST2H2AB(10);CENPB;ZFP30;STK38L;EIF5B;MKKS;CHUK;SNHG9;RPS28;LYRM7
	SIN3A ENCODE	4.5E-03	16	HIST1H2BK(11);DNAJC24;NARS;FAM172A;SLC25A40;RPL37;RRP15;POLH;EIF5B;SNHG9;MRPL30
GO Biological Process 2021	Cytoplasmic translation (GO:0002181)	1.5E-02	83	RPS28;RPS27;RPS29;RPL37A;RPL36A;TMA7;RPL38;RPL37;RPS21
	SRP-dependent cotranslational protein targeting to membrane (GO:0006614)	4.0E-02	63	RPS28;RPS27;RPS29;RPL37A;RPL36A;RPL38;RPL37;RPS21
	Cotranslational protein targeting to membrane (GO:0006613)	4.0E-02	59	RPS28;RPS27;RPS29;RPL37A;RPL36A;RPL38;RPL37;RPS21
KEGG 2021 Human	Ribosome	1.2E-02	40	MRPL30;RPS28;RPS27;RPS29;RPL37A;RPL36A;RPL38;RPL37;MRPL24;RPS21
	Herpes simplex virus 1 infection	1.2E-02	23	ZNF254;ZNF100;ZNF573;RNASEL;CHUK;PIK3R3;PIK3CD
	B cell receptor signaling pathway	1.2E-02	54	IFITM1;CHUK;PLCG2;PIK3CD;RAC3;PIK3R3;NFKBIE
	Inflammatory mediator regulation of TRP channels	3.1E-02	37	ADCY9;PLCG2;HTR2B;PIK3R3;PIK3CD;PRKACA;CALM2
WikiPathway 2021 Human	Histone Modifications WP2369	1.1E-15	1024	HIST1H3J(17)
Chromosome Location	chr6p22	7.7E-17	502	HIST1H2BO(24);SOX4;CAP2;RBM24

Enrichr Library	Term	Adj p_val(1)	Combined Score(1)	Representative Genes(2)
**Downregulated**				
ENCODE and ChEA Consensus TFs from ChIP-X	SUZ12 CHEA	1.0E-04	31	GALNT12;PCSK1;PTPRU;SPON1;NRP2;IL6R;SOX11;HOXC8;BCL11B;BCL11A
	SMAD4 CHEA	2.8E-02	18	PTPRU;NRP2;EDN1;LIMCH1;TMEM132B;CADPS;DHRS3;HS3ST2;PTPRD;FERMT1
	VDR CHEA	3.2E-02	40	CD274;CLMN;EPSTI1;ENTPD7;CYP19A1;N4BP2
MSigDB Hallmark 2020	KRAS Signaling Up	1.6E-05	82	IL33;SPON1;SEMA3B;G0S2;TNFRSF1B;EREG;BMP2;C3AR1;MAP7;SPP1;SCG5;SPRY2;KCNN4;TSPAN1;HBEGF
	Inflammatory Response	2.3E-04	54	EDN1;CSF3;TNFRSF9;PCDH7;AQP9;TNFRSF1B;EREG;ADORA2B;MYC;C3AR1;TNFSF9;BDKRB1;HBEGF
	TNF-alpha Signaling via NF-kB	2.5E-02	18	EDN1;BMP2;BCL2A1;MYC;TNFRSF9;TNFSF9;G0S2;PLPP3;HBEGF
	Estrogen Response Early	2.5E-02	18	SLC24A3;BCL11B;MYC;MYB;SEMA3B;DHRS3;THSD4;PTGES;TGM2
	Interferon Alpha Response	2.5E-02	25	HELZ2;CD74;RSAD2;OAS1;MX1;EPSTI1
	Wnt-beta Catenin Signaling	2.5E-02	39	HEY1;MYC;TCF7;DKK1
GO Biological Process 2021	Cytokine-mediated signaling pathway (GO:0019221)	6.2E-03	34	IL1RN;TNFRSF6B;IL24;RPS6KA5;MYC;ITGAX;CCL3;HMOX1;NCAM1;IL13RA2;IL6R;CCR1;IL33;RSAD2;TNFRSF9;MX1;TNFRSF1B;EREG;GREM2;AIM2;OAS1;TNFSF4;TNFSF9;CCL26
	Positive regulation of hormone secretion (GO:0046887)	6.2E-03	257	EDN1;NMB;SPP1;SOX11;CYP19A1
	Positive regulation of ERK1 and ERK2 cascade (GO:0070374)	6.2E-03	58	NECAB2;CCR1;CD74;BMP2;GAREM1;KDR;CCL3;CHI3L1;SPRY2;PTPN22;PRKCZ;CCL26
	Cellular response to cytokine stimulus (GO:0071345)	6.2E-03	35	CCR1;RIPOR2;IL1RN;CSF3;MME;IL24;TCF7;BRCA1;TNFRSF1B;EREG;GREM2;AIM2;OAS1;MYC;ITGAX;CCL3;HMOX1;CHI3L1;IL13RA2;IL6R;CCL26
	Regulation of ERK1 and ERK2 cascade (GO:0070372)	6.2E-03	47	NECAB2;CCR1;CD74;PTPN22;PRKCZ;BMP2;GAREM1;DUSP10;KDR;CCL3;CHI3L1;SPRY2;EPHB1;CCL26
	Positive regulation of type 2 immune response (GO:0002830)	1.2E-02	267	CD74;RSAD2;TNFSF4;PRKCZ
	Regulation of cell migration (GO:0030334)	1.2E-02	31	CCR1;NTNG1;PTPRU;CD74;EDN1;LIMCH1;SEMA3A;SEMA3B;FGF7;BMP2;DUSP10;KDR;PLXNA2;ITGAX;CCL3;SPRY2;HBEGF;CCL26
GO Cellular Component 2021	Integral component of plasma membrane (GO:0005887)	1.1E-02	20	PTPRU;PCDHGB6;SLC24A3;RDH8;GPR68;SEMA3A;AQP9;SEMA3B;ITPR3;ADGRG1
GO Molecular Function 2021	Receptor ligand activity (GO:0048018)	6.1E-03	41	IL33;NMB;EDN1;CSF3;SEMA3A;SEMA3B;GDF6;DKK1;EREG;GREM2;FGF7;BMP2;VGF;CCL3;HBEGF;CCL26
KEGG 2021 Human	Cytokine-cytokine receptor interaction	2.2E-03	45	CCR1;IL33;IL1RN;CSF3;TNFRSF6B;TNFRSF9;IL24;GDF6;TNFRSF1B;BMP2;TNFSF4;CCL3;TNFSF9;IL13RA2;IL6R;CCL26
Elsevier Pathway Collection	EGFR transactivation in Cancer and Non-Cancer Cells	3.6E-02	56	EDN1;NMB;MYC;BDKRB1;EREG;HBEGF

(1) – "Adjusted.P.value" and "Combined.Score" of Enrichr [19] gene sets analysis. The table includes only genes with an Adjusted.P.value < 0.05.

[2] – Representative transcripts from term-associated transcript sets. The list of histone gene transcripts is reduced to a single representative. The total number of histone genes is given in brackets, e.g., HIST1H2BO(17) means HIST1H2BO and 16 other histone gene transcripts.

Table S5. Mean and median contributions of viral transcripts to the transcriptome of cultured human brain cells 16 h after VV-GMCSF-Lact infection, with a statistical assessment of differences in mean viral transcript contributions between individual cell types.

**Cell Types**	**Contribution of viral transcripts**		**Significance of the differences in mean values Holm–Bonferroni adjusted t-test pVal***							
	Mean	Median	Mglv	OCv	Prcv	CLv	UNv	PNv	U87_1v	U87_2v
Fibv	6.99	0.78	4.2E-82	5.1E-09	3.3E-04	0.012	1.0	5.0E-30	1.0	0.23
Mglv	14.43	4.26		4.1E-110	3.6E-140	7.6E-24	3.1E-07	1.2E-04	1.2E-50	5.6E-77
OCv	3.74	0.17			0.040	1.0	0.019	6.9E-45	6.4E-05	4.0E-03
Prcv	5.23	0.78				0.59	0.32	6.1E-40	0.11	1.0
CLv	3.30	0.02					0.059	2.1E-26	0.051	0.23
UNv	7.73	0.17						2.9E-12	1.0	0.86
PNv	18.99	10.75							9.0E-29	5.4E-34
U87_1v	6.61	0.09								1.0
U87_2v	5.84	0.37								

* –Adjusted Holm-Bonferroni t-test p-values are presented in exponential form for statistically significant values (< 0.01) and in numerical form for values >= 0.01.

Note. The distribution of the relative contribution of viral transcripts according to the Shapiro-Wilk, Lilliefors, and Kolmogorov-Smirnov tests does not differ from the normal distribution at the level of $p < 0.05$ for all analyzed cell types.

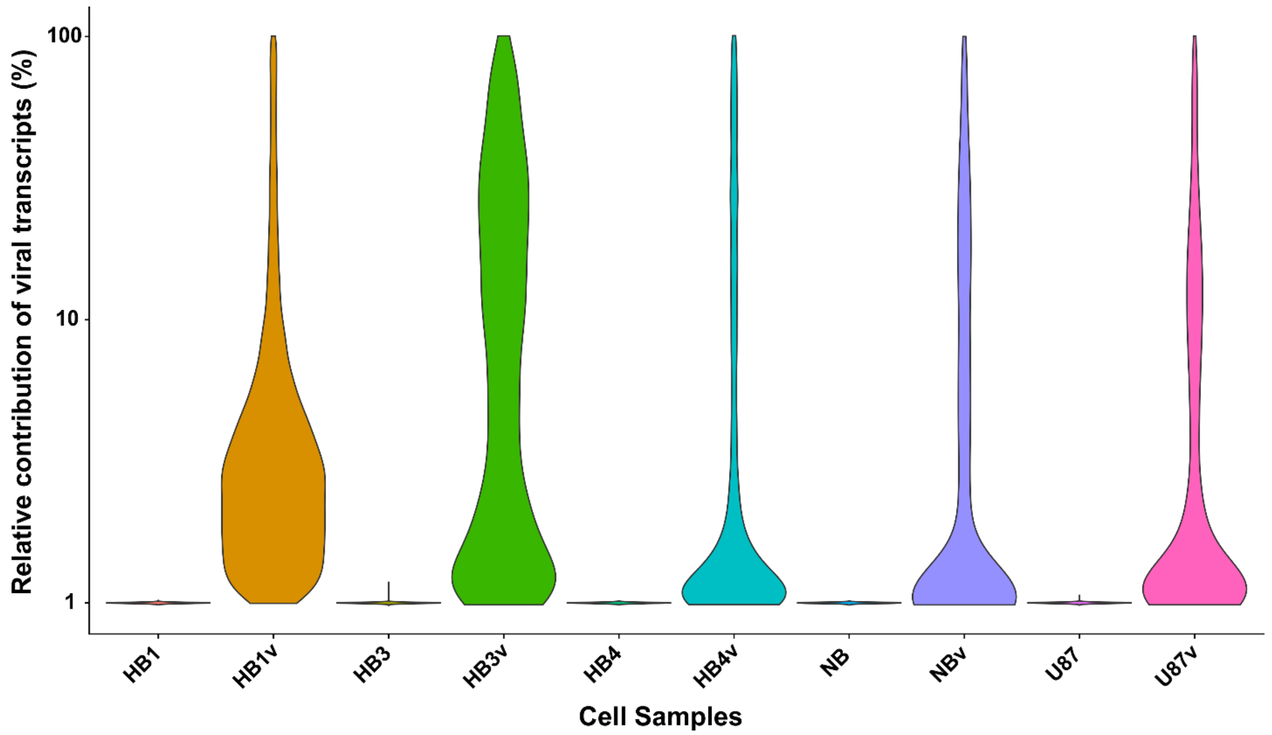

Figure S1. Violin plots showing the distribution of VV-GMCSF-Lact RNA as a proportion of total cellular RNA in analyzed cell cultures. The suffix "v" indicates cultures infected with VV-GMCSF-Lact (0.1 PFU/cell for 16 h).

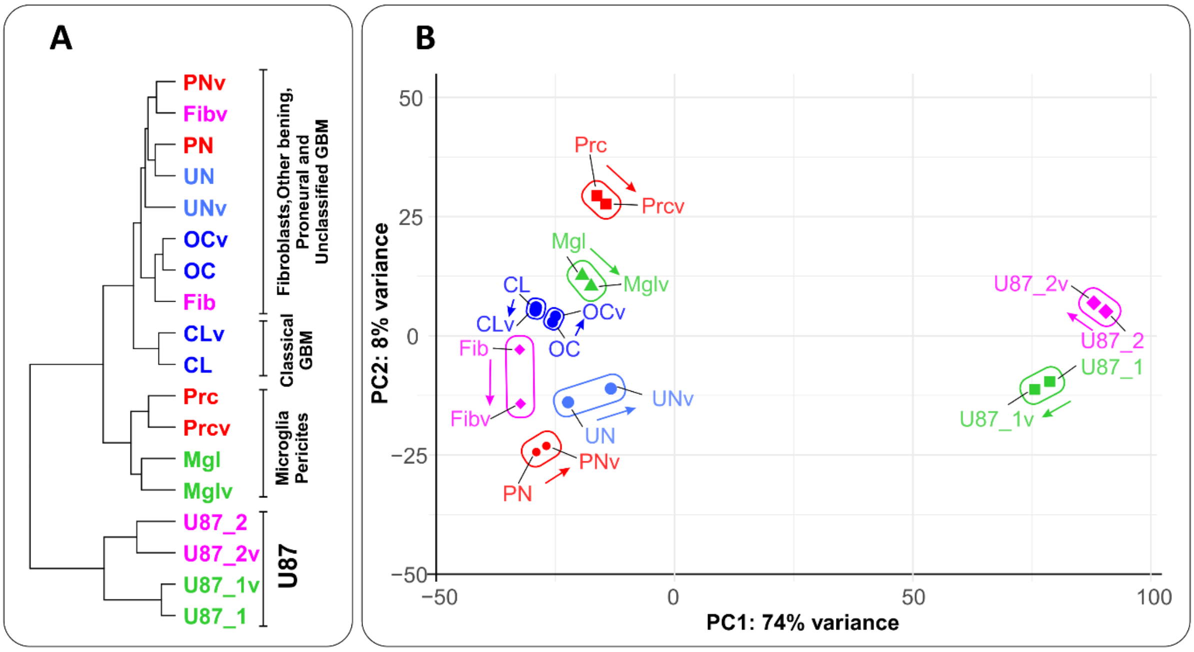

Figure S2. Hierarchical tree (A) and principal component analysis (B) of cell subtype transcriptomes. Aggregated single-cell RNA-seq data were used for the analysis. (A) The complete agglomeration method was used for clustering. (B) Principal component analysis of DESeq2 normalized VST-transformed aggregated RNA expression data. Sample-specific PC1:PC2 points are annotated with cell-defined envelopes. Arrows show trends in PC1 and PC2 changes between control and VV-GMCSF-Lact-infected cells of the same subtype. (A) and (B) use the same color scheme to indicate cell subtypes.

Note. When clustering single-cell RNA-seq data, U87 cells were found to form a distinct branch in the hierarchical tree, which was clearly divided into two subtypes designated as U87_1 and U87_2 (Figure S2, A). They also formed two distinct regions in the PCA plot, with a clear response to VV-GMCSF-Lact infection (Figure S2, B). Cells from patient-derived human brain cultures form a separate branch divided into subclusters of Prc and Mgl, GBM CL, as well as a subcluster of Fib, OC with PN, and GBM UC. The corresponding PCA plot confirms that Prc and Mgl cells are distant from OC, Fib, GBM PN, CL, and UN, and show a similar trend when infected with the virus in PC1:PC2 coordinates (Figure S2, B).

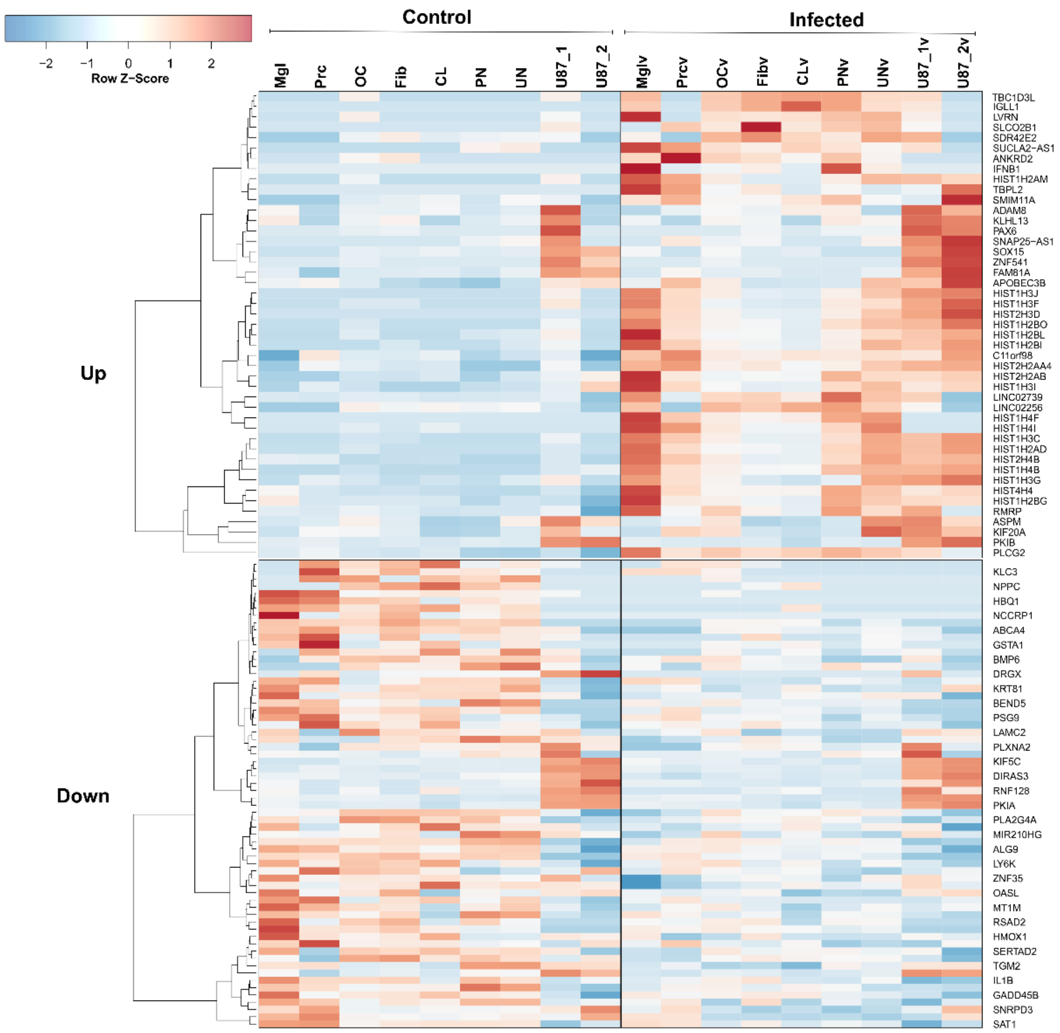

Figure S3. Heatmap showing representative sets of the top 50 upregulated and top 50 downregulated transcripts in VV-GMCSF-Lact-infected human brain cell cultures, aggregated by cell types and normalized with DeSeq2 after VST transformation of raw sequencing data.

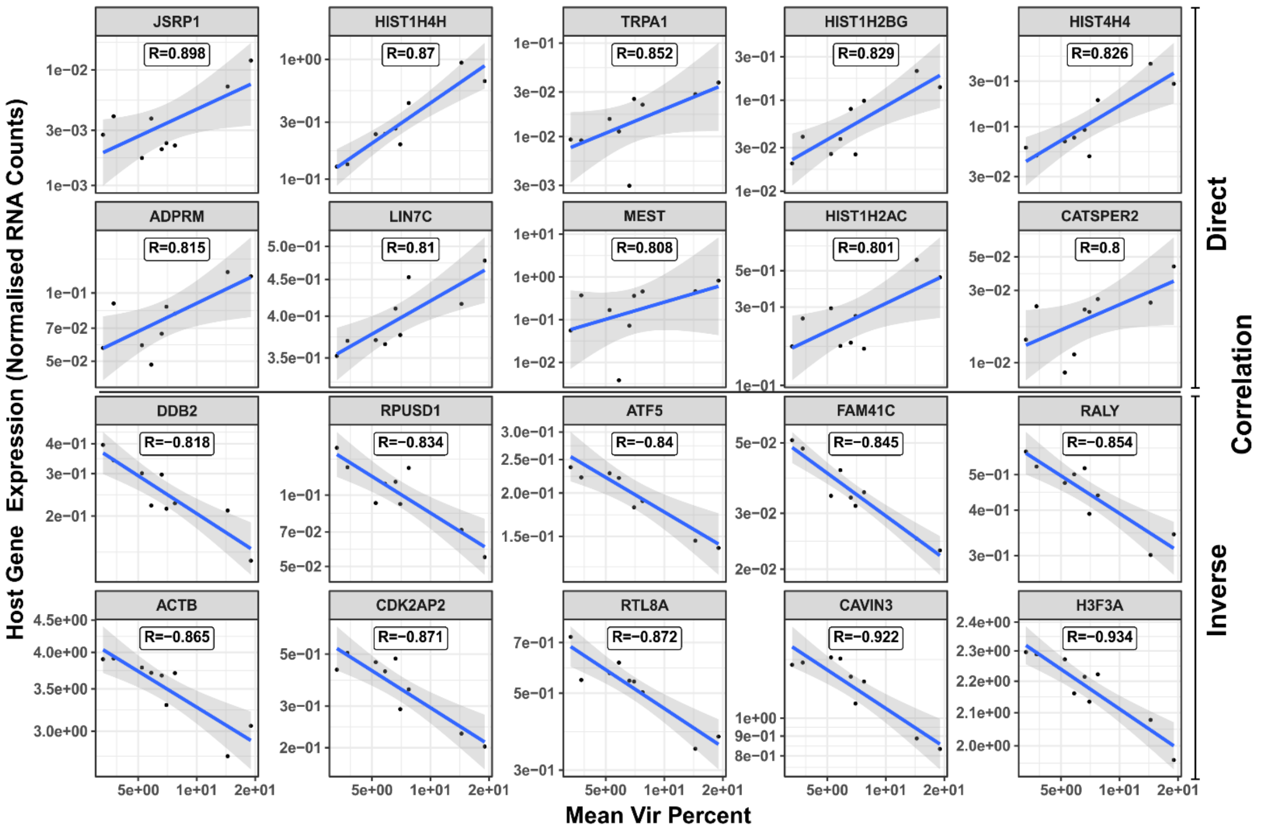

Figure S4. Dot plots representing the correlation of selected human transcripts with the mean contribution of viral RNA to the cellular transcriptome in a panel of VV-GMCSF-Lact-infected human brain cells. The X-axis displays the susceptibility of identified brain cell types to VV-GMCSF-Lact infection in terms of the mean relative contribution of viral transcripts (%) in the order PNv > Mglv >> UNv > Fibv > U87_1v > U87_2v > Prcv >> OCv > CLv, as described in Section 3.4 and presented in Table S5. The Y-axis displays normalized RNA counts for specific gene transcripts. R – Pearson's correlation coefficient.

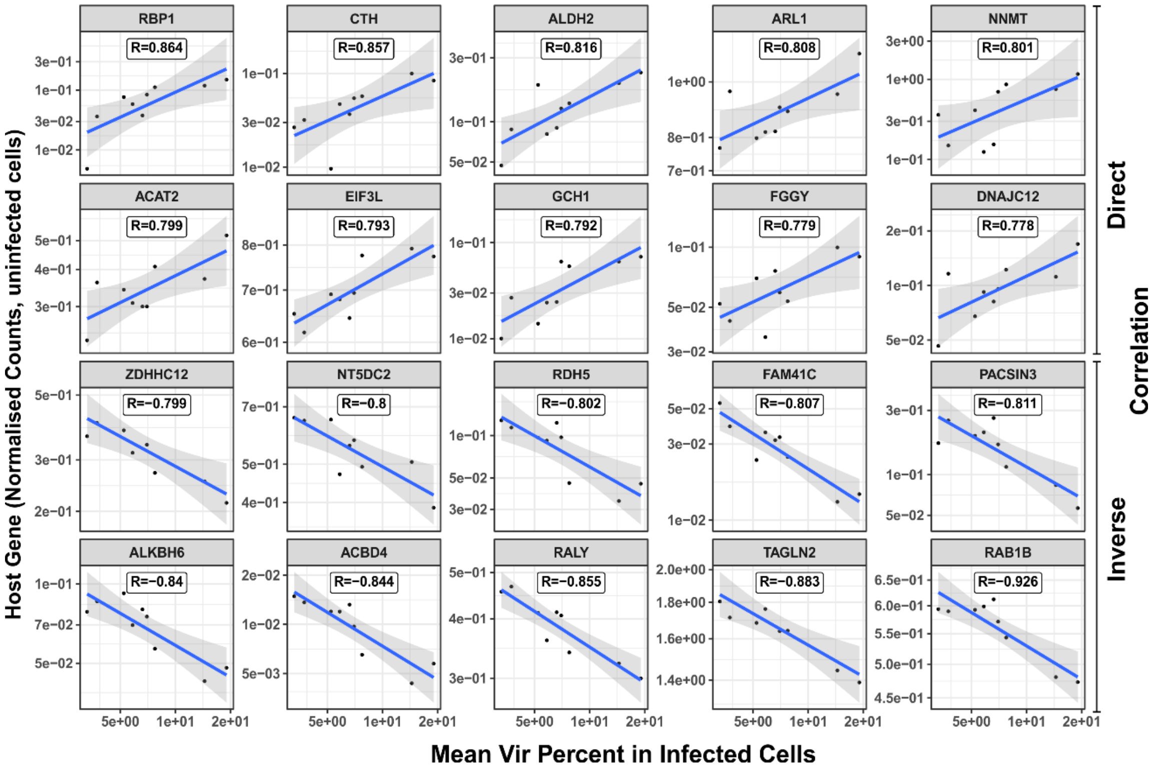

Figure S5. Dot plots representing the correlation of selected human transcripts with the mean contribution of viral RNA to the cellular transcriptome in a panel of uninfected human brain cells. R – Pearson's correlation coefficient. The x-axis displays the susceptibility of identified brain cell types to VV-GMCSF-Lact infection in terms of the mean relative contribution of viral transcripts (%) in the order PNv > Mglv >> UNv > Fibv > U87_1v > U87_2v > Prcv >> OCv > CLv, as described in Section 3.4 and presented in Table S5. The Y-axis displays normalized RNA counts for specific gene transcripts. R – Pearson's correlation coefficient.

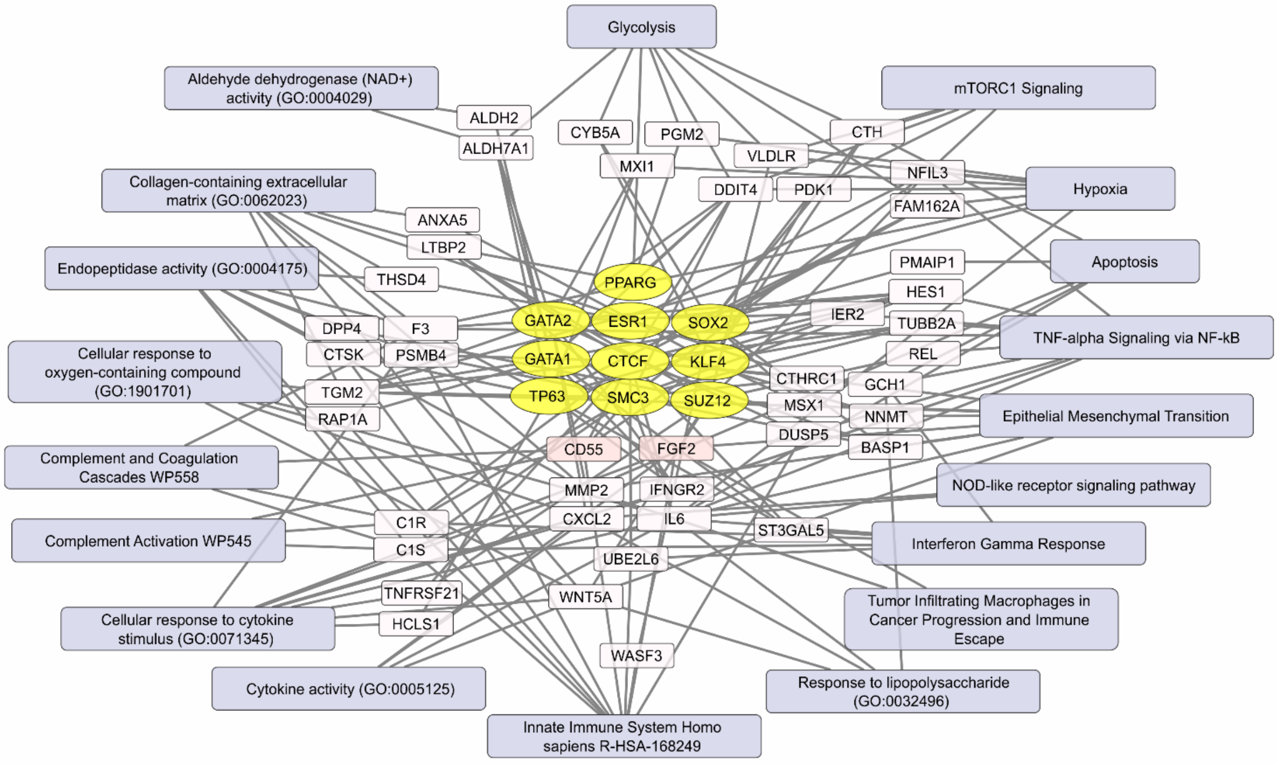

Figure S6. Selected networks of transcription factors, gene products, and processes derived from the list of transcripts positively correlated ($R > 0.5$) with the mean contribution of VV-GMCSF-Lact RNA in uninfected cells. Transcription factors are shown in yellow ovals; selected activated genes — in white and pink rectangles; signaling pathways, biological processes, and gene annotations — in light gray-blue. Transcripts CD55 and FGF2, which are commonly upregulated in infected cells and are linked to the sensitization of uninfected cells to the virus, are shown in pink rectangles. The following Enrichr libraries were used to analyze the top 300 genes: "ENCODE and ChEA Consensus TFs from ChIP-X"; "MSigDB Hallmark 285 2020"; "GO Biologic Process 2021"; "Panther 2016"; "KEGG 2021 Human" and "Chromosome Location hg19".

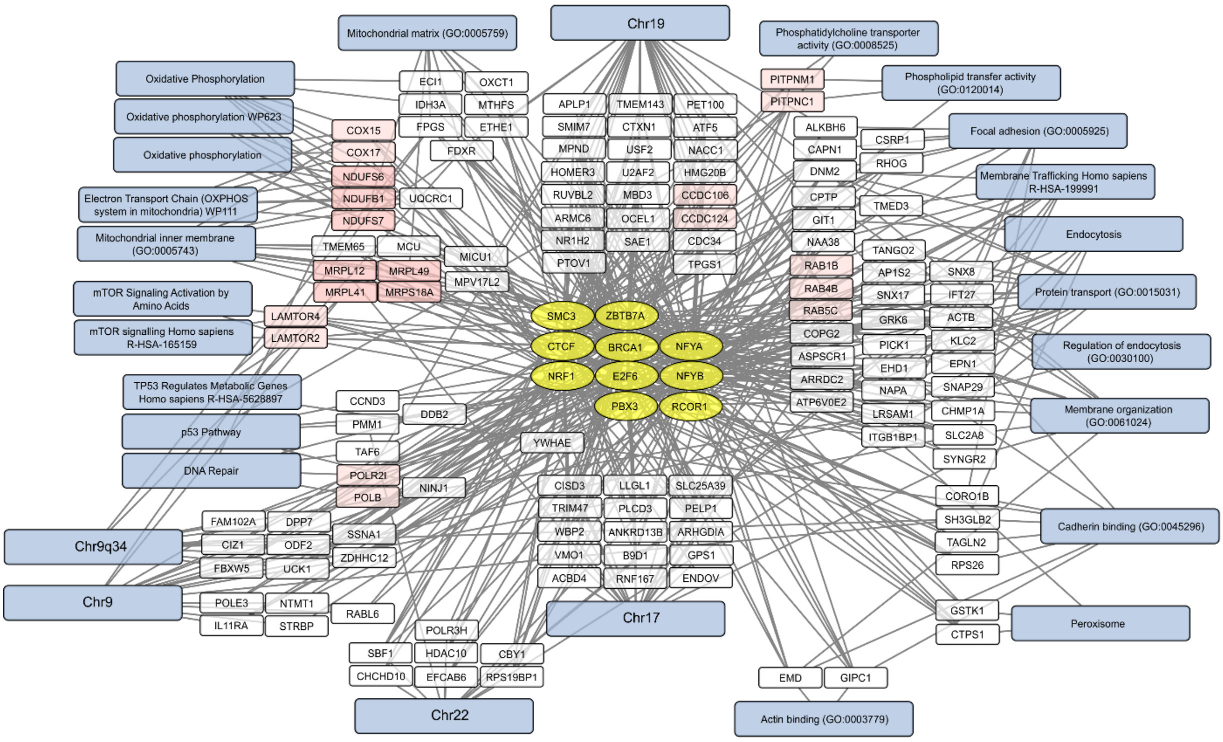

Figure S7. Selected networks of transcription factors, gene products, and cellular processes derived from the list of transcripts negatively correlated (R < -0.5) with the mean contribution of VV-GMCSF-Lact RNA in uninfected cells. Transcription factors are shown in yellow ovals; selected activated genes — in white and pink rectangles; signaling pathways, biological processes, and gene annotations — in light gray-blue. Genes with similar functions are grouped and colored in pink. The following Enrichr libraries were used to analyze the top 300 genes: "ENCODE and ChEA Consensus TFs from ChIP-X"; "MSigDB Hallmark 285 2020"; "GO Biologic Process 2021"; "Panther 2016"; "KEGG 2021 Human" and "Chromosome Location hg19".

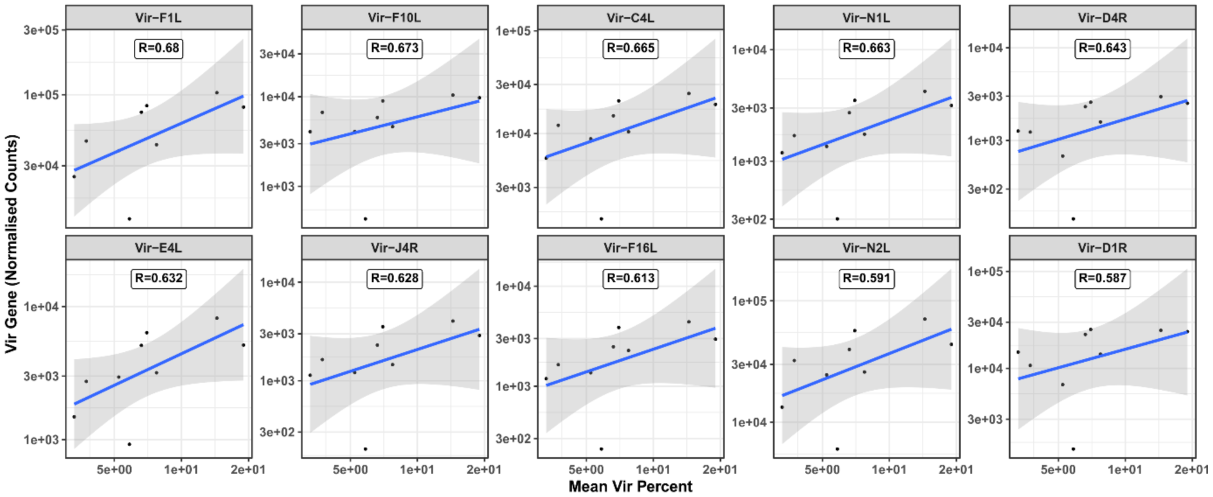

Figure S8. Dot plots representing individual VV-GMCSF-Lact transcripts that directly correlate (R > 0.5) with viral RNA percent in cultured human brain cells. R — Pearson correlation coefficient. The X-axis displays the susceptibility of identified brain cell types to VV-GMCSF-Lact infection in terms of the mean relative contribution of viral transcripts (%) in the order PNv > Mglv >> UNv > Fibv > U87_1v > U87_2v > Prcv >> OCv > CLv, as described in Section 3.4 and presented in Table S5. The Y-axis displays normalized RNA counts for specific VV-GMCSF-Lact transcripts. R – Pearson's correlation coefficient.
